# Perceived professional benefits among nursing interns in the later stage of clinical practicum: a descriptive qualitative study

**DOI:** 10.3389/fmed.2026.1946813

**Published:** 2026-09-24

**Authors:** Sixing Liu, Fengying Lin, Caihong Xue, Lihua Chen, Yuanfeng Lv, Fan Wei, Chaoluan Rao, Xiaojing Chen, Minxiang Li

**Affiliations:** 1 Department of Nursing, The Second Affiliated Hospital of Hainan Medical University, Haikou, Hainan, China; 2 School of Public Health, Kunming Medical University, Kunming, Yunnan, China

**Keywords:** clinical practicum, nursing interns, nursing workforce retention, perceived professional benefits, qualitative study

## Abstract

**Background:**

The later stage of clinical practicum is a critical period during which nursing interns transition from the role of students to that of registered nurses. Their perceived professional benefits are closely associated with their willingness to enter the nursing profession.

**Objective:**

This study explores perceived professional benefits among nursing interns during the later stage of their clinical practicum, aiming to provide guidance for optimizing teaching strategies at this stage.

**Methods:**

A descriptive qualitative study design was adopted. Semi-structured interviews were conducted with 12 nursing interns in the later stage of their clinical practicum at a tertiary Grade A comprehensive teaching hospital in Hainan Province. The participants comprised 7 students from a four-year baccalaureate program (including one with a bachelor's degree upgraded from junior college) and 5 students from a three-year junior college program. Thematic analysis was used for data analysis, and NVivo 12.0 software was employed to assist with data management.

**Results:**

This study identified 4 themes: obtaining diverse support, strengthening clinical practice competence, perceiving professional rewards, and deepening conviction. Nursing interns conducted clinical practicum in a harmonious departmental atmosphere and received guidance and care from preceptors, as well as support from family and friends. As clinical practicum progressed to the later stage, nursing interns improved their clinical operation skills, practical confidence, and comprehensive professional literacy. They also perceived professional advantages, gained recognition from others, and experienced the value of using professional knowledge to help family and friends. These positive experiences continuously strengthen the nursing interns' professional conviction while simultaneously enhancing their perceived professional benefits.

**Conclusions:**

The later stage of clinical practicum is a critical period for the formation of nursing interns' perceived professional benefits. Hospitals should create a supportive practicum atmosphere and strengthen positive incentives to enhance nursing interns' perceived professional benefits, which can help facilitate their smooth transition to registered nurses.

## 1 Introduction

The World Health Organization, in its *Global Strategic Directions for Nursing and Midwifery*, proposes that nursing education should be competence-oriented and ensure that students obtain adequate pre-service clinical practicum opportunities (1). In China, baccalaureate nursing programs are four years in length, while junior college programs are three years, both of which include three components: general education courses, professional courses, and clinical practicum (2). Despite differences between the two educational levels, both require nursing interns to complete a specified duration of clinical practicum before they can graduate and take the national nursing licensure examination (3). Specifically, junior college nursing interns are generally required to complete no less than 8 months of clinical practicum, whereas baccalaureate nursing interns are generally required to complete no less than 10 months (4). It is evident, therefore, that clinical practicum is a critical stage during which nursing interns transition from students to licensed nurses.

Clinical practicum is a gradual and dynamic process, and the practicum period can be divided into three stages: early, middle, and late (5). Yang et al. (6) define the later stage of the practicum as the final 1–3 months before its conclusion. Nursing interns in the later stage of their practicum have generally completed rotations through various departments and may have accumulated some experience in clinical procedures, patient observation, communication and collaboration, and nursing documentation. They are capable of performing routine clinical nursing tasks under the guidance of a preceptor (7).

At the same time, nursing interns must simultaneously cope with multiple pressures, such as exams and career choices (8). If the transition during this stage is not smooth, nursing interns are prone to experiencing transition shock, developing negative emotions, and facing increased uncertainty about their future careers. Some may even abandon the nursing profession when choosing a career path, resulting in the loss of prospective nursing talent (9). Therefore, the later stage of clinical practicum is a period in which nursing interns' clinical competencies tend to mature while their professional attitudes are prone to wavering. Their authentic professional experiences warrant close attention.

Among various professional experiences, perceived professional benefits in nursing are a cognitive evaluation grounded in positive psychology. They refer to the benefits and gains that nursing staff perceive in clinical practice, along with their recognition that the profession contributes to their comprehensive development (10). These benefits encompass multiple dimensions, including material rewards, professional growth, and social recognition (11). When nursing interns perceive higher levels of perceived professional benefits, they tend to develop stronger confidence and enthusiasm toward the nursing profession (11).

It is worth noting that perceived professional benefits are related to concepts such as clinical self-efficacy, professional identity, job satisfaction, and career intention, yet they differ in their conceptual connotations. Specifically, clinical self-efficacy emphasizes individuals' subjective confidence in their own theoretical knowledge and operational skills (12). Professional identity focuses on individuals' sense of belonging to and internalization of the nursing professional role (13). Job satisfaction refers to individuals' immediate satisfaction with specific work environments and conditions (14). Career intention reflects individuals' willingness to enter and remain in the nursing profession after graduation (15). In contrast, perceived professional benefits are not a unidimensional assessment of any single aspect, but rather a holistic perception of the positive experiences and personal growth significance brought by the profession.

In recent years, perceived professional benefits have attracted widespread attention due to their positive effects in alleviating job burnout and stimulating enthusiasm for the nursing profession (16). Existing studies on nursing interns' perceived professional benefits have predominantly relied on quantitative methods, focusing on cross-sectional surveys and analyses of influencing factors. Such approaches fail to deeply capture the authentic inner experiences of nursing interns during the later stage of clinical practicum, a uniquely critical period. To address this gap, the present study adopts a descriptive qualitative research design. Semi-structured interviews were employed to gain an in-depth understanding of nursing interns' perceived professional benefits during the later stage of clinical practicum. The findings are intended to inform strategies for optimizing clinical practicum teaching, enhancing nursing interns' perceived professional benefits, and stabilizing the nursing workforce.

## 2 Methods

### 2.1 Study design

This study adopted a descriptive qualitative research design. Grounded in naturalistic inquiry, this design emphasizes presenting findings with minimal theoretical interpretation, keeping themes and descriptions close to participants' original expressions. It is suitable for research questions where understanding of a phenomenon is limited and where direct access to the experiences and perspectives of those involved is needed (17). Perceived professional benefits are a subjective experience of nursing interns in the context of clinical practicum, characterized by distinct individualized and contextual features. This study aims to obtain direct descriptions and authentic expressions of perceived professional benefits from nursing interns during the later stage of clinical practicum. There is no need for deep theoretical abstraction or construction of the data. Therefore, the descriptive qualitative design aligns well with the purpose of this study. To ensure transparency and rigor in the data collection and analysis process, this study adheres to the Consolidated Criteria for Reporting Qualitative Research (COREQ) (18).

### 2.2 Sample and setting

The study was conducted from January to February 2025 at a tertiary Grade A comprehensive teaching hospital in Hainan Province. Hospitals in China operate under a three-tier accreditation system, with “tertiary Grade A” representing the highest level in the system. This designation denotes a large, comprehensive hospital with extensive scale, a full range of medical disciplines, and responsibilities in medical care, teaching, and research. As a university-affiliated hospital, this institution is responsible for the region's primary clinical nursing education, recruiting approximately 220 nursing interns annually. These interns include baccalaureate and junior college students from collaborating institutions within the province through unified placement, along with some students from outside the province who independently arrange their clinical practicum at this hospital. Meanwhile, Nursing interns are assigned to various departments for rotations by the Nursing Department, with each rotation supervised on a one-on-one basis by a registered nurse qualified as a preceptor.

The researchers verified through the hospital's clinical placement system that the nursing interns' practicum completion dates were concentrated between March and April. Given that the study was conducted from January to February, the nursing interns were in the final 1–3 months of their practicum and had entered the later stage of clinical practicum. Subsequently, the researchers contacted the teacher responsible for practicum management in the Nursing Department, explained the purpose of the study, and obtained permission. After the teacher provided a list of nursing interns currently in the later stage of practicum, the first author (L) used purposive sampling to conduct a preliminary screening based on the inclusion and exclusion criteria. After identifying potential participants, the researcher L met with them in person to explain the purpose of the study, the interview content, confidentiality principles, and their rights to voluntary participation and withdrawal at any time. After obtaining their consent and having them sign an informed consent form, the researcher scheduled the time and location for the interview.

The inclusion criteria were as follows: ① undergraduate or vocational nursing interns who have completed all nursing coursework; ② were in the final 1–3 months of their practicum; and ③ provide informed consent and voluntarily participate in this study. The exclusion criteria were as follows: ① those with severe mental or psychological issues that prevent them from cooperating with the study; and ② nursing interns who have been returned to their school by the hospital due to violation of clinical practicum regulations. The sample size for this study was determined based on the principle of data saturation, which means that no new themes or perspectives emerge during data collection and information redundancy begins to appear (19). Data saturation was achieved after conducting interviews with 10 nursing interns, and two supplementary interviews were subsequently conducted to verify this, with no new data generated. Therefore, a total of 12 nursing interns were ultimately included in this study.

### 2.3 Data collection

#### 2.3.1 Interview outline

Based on the research purpose, the researchers reviewed the literature (20, 21) and developed the interview outline through discussion with the team members. The interview outline was developed in Chinese, and all interviews were conducted in Chinese. Prior to the formal interviews, one baccalaureate nursing intern and one junior college nursing intern were selected for pilot interviews, and their interview data were not included in the outcome analysis. After the pilot interviews, the outline was further refined through discussion with the team to ensure its relevance and applicability, and the final version of the interview outline was formed (Table 1).

**Table 1:** Final version of the interview outline.

Main question
1. Do you have a sense of identification with the nursing profession? Has your clinical practicum experience enhanced your perceived professional benefits?
2. During your clinical practicum, what experiences have given you a positive view of the nursing profession? Could you describe these experiences in detail?
3. How are your relationships with your clinical instructors and peers in the department? Do you feel a sense of belonging to the team? What specific effects do these interpersonal interactions have on your perceived professional benefits?
4. During your clinical practicum, have there been any experiences that made you feel support and recognition from your family and friends? Do these support and recognition have an impact on your perceived professional benefits?
5. In the process of interacting with patients, what experiences have given you a particular sense of achievement? Please describe in detail how these experiences have influenced your perceived professional benefits.
6. During your clinical practicum, what experiences have made you aware of your deficiencies in professional skills or overall competence, and thus prompted you to work on improvement?
7. During your clinical practicum, in what areas do you feel you have achieved significant growth or improvement? Do these have an impact on your future career development?
8. Apart from the aspects mentioned above, what other factors do you think may affect your perceived professional benefits?

#### 2.3.2 Interview process

This study employed a semi-structured interview method to conduct one-on-one in-depth interviews with nursing interns. All interviews were conducted independently by the researcher L, who is a master's student in nursing at the hospital. Prior to the interviews, researcher L had no prior acquaintance with the participants and did not participate in their clinical instruction, supervision, or any form of evaluation. The interview results were not associated with the participants' practicum performance or graduation assessments. The interviewer, drawing on her professional background, recruited and contacted nursing interns who met the inclusion criteria through the clinical placement coordinator. Prior to the interviews, the interviewers provided the nursing interns with a detailed explanation of the study purpose, emphasized that there were no right or wrong answers, and assured them that all interview data would be strictly encrypted and anonymized through coding rather than names. They also informed the participants that they could withdraw at any time without affecting their clinical practicum evaluations. The interviews were initiated only after obtaining informed consent.

The interviews were conducted in private and quiet settings, such as demonstration classrooms or rest rooms, with the timing scheduled to avoid peak clinical practicum hours, so as to ensure privacy and comfort during the conversations. During the interviews, the interviewers followed the semi-structured interview outline, employed empathy and listening skills, and maintained a neutral stance. They avoided leading questions and non-verbal cues, and encouraged the nursing interns to share their experiences and feelings honestly and thoroughly. Researcher L was similar in age to the participants, which helped establish a rapport based on equality and trust and encouraged them to express themselves authentically. Each interview lasted approximately 20–40 min, and with the participants' consent, the entire session was audio-recorded, while non-verbal information was also documented. Within 24 h after the interviews, the researchers promptly transcribed the audio recordings to ensure that the information was complete and accurate.

#### 2.3.3 Translation and verification of interview data

All interviews were conducted in Chinese, and the audio recordings were transcribed verbatim in Chinese for analysis. The quotations presented in this manuscript were translated into English through a rigorous translation and verification process. First, researcher L, a native Chinese speaker with proficiency in English academic writing, translated the selected quotations from Chinese to English. Second, to ensure conceptual equivalence and preserve the original meaning of participants' expressions, another researcher (corresponding author L), who has extensive experience in qualitative research and English academic writing, independently reviewed the translations against the original Chinese transcripts. Any disagreements regarding terminology, wording, or nuanced meanings were discussed between the two researchers until consensus was reached. Through this combined process of forward translation and independent verification, we ensured that the English quotations faithfully retain the original expressions of the nursing interns.

### 2.4 Data analysis

In this study, data collection and data analysis were conducted concurrently. After each interview, transcription and preliminary analysis were initiated immediately, and the analysis findings were used to guide subsequent interviews and to determine data saturation. NVivo 12.0 software was used to assist with data management, coding, and analysis.

This study adopted an inductive thematic analysis approach, following the six-phase framework proposed by Braun and Clarke (22) in 2006. The inductive path was chosen to allow themes to emerge from the participants' original narratives rather than being imposed by a preconceived theoretical framework, which aligns with the nature of descriptive qualitative research. Two researchers independently read all transcripts and generated initial codes through open coding by extracting meaning units line by line from the original data. They then compared and discussed their respective codes, categorized and integrated related codes, and gradually developed subthemes and themes through iterative discussion and refinement. When disagreements arose regarding code definitions or theme boundaries, a third researcher was involved in the discussion to facilitate consensus.

The specific steps were as follows: ① Familiarising with data: all interviews were transcribed verbatim within 24 h after each interview. The researchers read the transcripts repeatedly, immersed themselves in the data to gain a holistic understanding, and recorded preliminary analytical thoughts. ② Generating initial codes: the researchers read all transcripts line by line using open coding, extracting meaning units relevant to the research questions from the original data to generate initial codes. This process did not presuppose any pre-existing coding framework, and all codes were derived directly from participants' own words. Subsequently, the data extracts corresponding to each code were systematically organized. ③ Searching for themes: codes with similar meanings were categorized and integrated, first forming subthemes and then further consolidating them into candidate themes, while compiling all data extracts relevant to each candidate theme. ④ Reviewing themes: candidate themes were checked against the coded extracts and the entire dataset to ensure consistency. Through repeated comparison and refinement, a thematic framework was developed. ⑤ Defining and naming themes: the content of each theme was further refined, and each theme was clearly defined, named, and described in detail. ⑥ Producing the report: representative and vivid data extracts were selected to support each theme, and the findings were linked to the research questions and relevant literature to produce the final report. The analytical process was not linear but rather iterative, with constant returning to the data. After the analysis was completed, the thematic results were returned to the nursing interns for verification, and the final findings were confirmed.

### 2.5 Rigor of study

To ensure the rigor of the study and the trustworthiness of the findings, this study adhered to the criteria of credibility, dependability, confirmability, and transferability proposed by Lincoln and Guba (23). Regarding credibility, the interviewer received systematic training in qualitative research courses prior to the interviews. During the interviews, she focused primarily on listening, avoided subjective guidance, and recorded participants' body language and vocal tones. After the preliminary themes were developed, the researchers sent the theme names, definitions, and representative quotes back to all 12 nursing interns individually via WeChat for verification. They were asked to judge whether the themes accurately reflected their authentic experiences. All 12 nursing interns confirmed that the thematic content was consistent with their own feelings, endorsed the analytical findings, and proposed no modifications. Regarding dependability, data analysis and coding were independently performed by two researchers. In cases of disagreement, a third researcher was involved for discussion until consensus was reached. Regarding confirmability, the research team held regular thematic coding meetings, and the researchers wrote reflective journals promptly after each interview. Regarding transferability, this study provided a detailed description of the research setting characteristics, including the hospital grade and the clinical practicum supervision model, as well as demographic information such as the nursing interns' educational levels and the number of rotation departments. The specific implementation process of sampling was also clearly reported, enabling readers to assess the potential for transferring the findings to other contexts.

## 3 Results

### 3.1 Students' characteristics

Among the 12 nursing interns, 4 were male and 8 were female. Their ages ranged from 20 to 24 years, with a mean age of 21.92 ± 1.31 years. Six were baccalaureate nursing interns, one had a bachelor's degree upgraded from junior college, and five were junior college nursing interns. Most nursing interns chose the nursing profession primarily due to employment prospects or personal interest, and the majority expressed a general intention to continue working in nursing after graduation. In addition, due to differences in practicum schedules across institutions, the start and end dates of clinical practicum and the number of rotated departments varied among the nursing interns. However, all met the basic requirement that junior college interns complete no less than 8 months of practicum and baccalaureate interns no less than 10 months. The demographic and other relevant characteristics of the nursing interns are presented in Table 2.

**Table 2:** Demographic and practicum-related characteristics of the nursing interns.

No.	Age (years)	Gender	Education	Planned clinical rotations (n)	Total clinical rotations experienced (n)	Remaining practicum weeks (weeks)	Reason for choosing nursing	Post-graduation career intention
S1	22	Female	Baccalaureate	10	8	Approximately 7 weeks	Parents' or relatives' choice	Continue working in nursing
S2	20	Male	Junior college	11	9	Approximately 7 weeks	Employment prospects	Continue working in nursing
S3	20	Female	Junior college	9	7	Approximately 7 weeks	Employment prospects	Continue working in nursing
S4	22	Male	Baccalaureate	10	8	Approximately 7 weeks	College admission reassignment	Continue working in nursing
S5	24	Female	Junior college	11	9	Approximately 7 weeks	Personal interest	Further study in nursing
S6	23	Female	Bachelor's (upgraded from junior college)	10	8	Approximately 7 weeks	Employment prospects	Further study in nursing
S7	23	Male	Baccalaureate	10	9	Approximately 7 weeks	College admission reassignment	Continue working in nursing
S8	22	Male	Junior college	11	9	Approximately 7 weeks	Employment prospects	Further study in nursing
S9	20	Female	Junior college	11	9	Approximately 9 weeks	Personal interest	Continue working in nursing
S10	22	Female	Baccalaureate	10	9	Approximately 4 weeks	College admission reassignment	Continue working in nursing
S11	22	Female	Baccalaureate	10	9	Approximately 4 weeks	Employment prospects	Continue working in nursing
S12	23	Female	Baccalaureate	9	7	Approximately 10 weeks	Personal interest	Continue working in nursing

### 3.2 Themes

This study identified 4 main themes: obtain diverse support, strengthen clinical practice competence, perceive professional rewards, and deepen professional conviction. In addition, 12 subthemes were extracted, as detailed in Figure 1.

**Figure 1:**
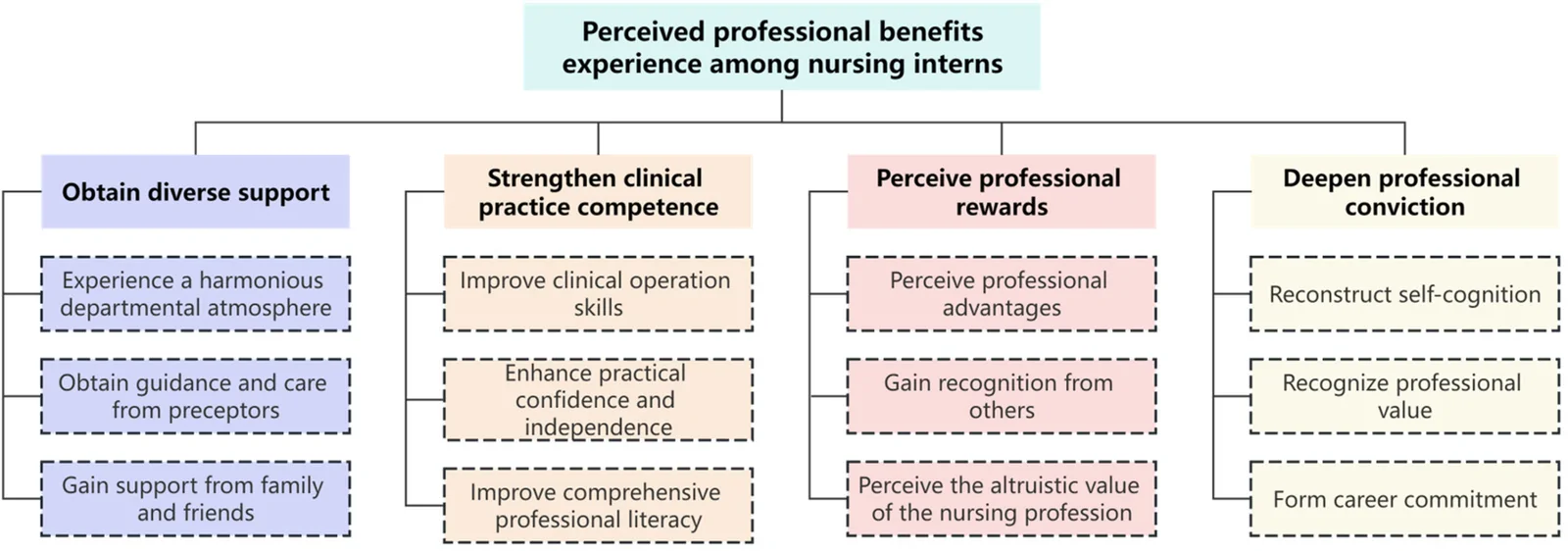
Optimized thematic map of themes and subthemes.

#### 3.2.1 Obtain diverse support

During clinical practicum, nursing interns do not face clinical challenges in isolation. Instead, they receive support from multiple sources, including the departmental atmosphere, preceptors, and family and friends. This support encompasses both work-related collaboration and guidance, as well as emotional care and encouragement. Diverse support not only helps alleviate nursing interns' practicum-related stress but also contributes to enhancing their clinical practice competence.

##### 3.2.1.1 Experience a harmonious departmental atmosphere

Nursing interns report that the departmental working atmosphere can affect their work efficiency and clinical practicum experience. A harmonious departmental atmosphere and mutual support from colleagues can not only improve work efficiency but also enhance a sense of team belonging and workplace well-being. A positive and harmonious departmental atmosphere not only brings nursing interns individual positive emotions but also facilitates a collaborative group atmosphere.

“I had the best clinical practicum experience in the ICU. The preceptors in the department would help each other out by sharing the workload. When my preceptor's own group was not busy, she would take me along to help colleagues directly. Compared to being sent on errands alone and feeling at a loss, I much preferred it when my preceptor involved me in team collaboration.” (S4)

“The atmosphere in a department has a significant impact on nursing efficiency. A poor atmosphere can lead to internal friction and delays in work. In departments where relationships are harmonious, colleagues actively help one another and work together to complete tasks, resulting in significantly higher efficiency.” (S9)

##### 3.2.1.2 Obtain guidance and care from preceptors

The individualized guidance and positive teaching attitude of preceptors are key elements of clinical teaching, which can effectively promote the development of nursing interns' practical competence. Nursing interns reported that preceptors followed the principle of “being hands-off but keeping eyes on”—allowing students to perform procedures independently while maintaining close supervision. Preceptors proactively created opportunities for nursing interns to practice, provided detailed and targeted instruction on weak skills, helped them overcome operational difficulties, and consolidated their clinical skills through repeated practice.

“I learned how to draw blood gas samples in the emergency department … When I first arrived, I mentioned that I'd tried it before but never succeeded. My instructor said, ‘As long as you’re here, I’ll make sure you learn how to do it.’ He teaches very thoroughly, and now I can usually get the needle in, unless the vein is particularly hard to access.” (S8)

“When I was in the gynecology department, my instructor was really great. Whenever there was work to be done, we’d do it together. For procedures like inserting IV catheters, she’d take me along right away. She’d say, ‘Whenever there's a new procedure, let the nursing interns try it—if you don't practice, you'll never learn.’” (S10)

In addition to teaching professional skills, preceptors also provide important emotional care and psychological support. When nursing interns encounter setbacks in clinical practice, preceptors offer timely emotional counseling.

“I had a good relationship with several preceptors. Once, a typhoon was approaching, and my preceptor told me not to come to work. I said no and insisted on going with her because I really wanted to do my part. She treated me so well—whenever I worked with her, she would buy me meals and milk tea.” (S8)

“I was once criticized by a doctor in the operating room. I worried about letting my preceptor down, so I apologized to her. She comforted me instead and told me, ‘It’s common for junior nurses to be scolded—the most important thing is to stay calm and positive.’” (S10)

##### 3.2.1.3 Gain support from family and friends

The emotional companionship and empathetic understanding from peers, along with the encouragement and guidance from family members regarding academic planning and career development, constitute important sources of social support for nursing interns. These supports help buffer various pressures arising from clinical practicum and sustain nursing interns' intrinsic motivation for clinical learning and work.

“I talk to my friends about frustrating things, and they vent along with me. My family is also very supportive—they often encourage me and are very understanding. But I tend to share negative emotions more with my friends, because peers are more empathetic.” (S1)

“My parents are very supportive of my choices. For example, if I want to take the postgraduate entrance exam—even if I don’t pass or take time off work—my family will still support me. My sister is also a graduate student. I see her as a role model, and I want to follow in her footsteps.” (S6)

#### 3.2.2 Strengthen clinical practice competence

During the later stage of clinical practicum, nursing interns' clinical practice competence improves notably in terms of operational skills, practical confidence and independence, and comprehensive professional literacy. Nursing interns gradually transition from relying on preceptors to being able to independently complete some or all nursing tasks, and they enhance their communication and collaboration skills in diverse clinical situations.

##### 3.2.2.1 Improve clinical operation skills

During the later stage of clinical practicum, nursing interns show significant improvement in multiple basic nursing and specialized clinical operation skills. Through repeated practice, they achieve proficiency, and the skills they have acquired lay a practical foundation for their future clinical work.

“During the later stage of clinical practicum, I improved a lot in many procedures, for example, urinary catheterization, skin preparation, inserting a disposable steel needle, and using infusion pumps.” (S2)

“I learned a lot of specialized procedures in the neurology department, such as the water swallow test, muscle strength assessment, heart rate variability analysis, and sleep quality monitoring. These will definitely be useful in my future work. Practice makes perfect.” (S3)

##### 3.2.2.2 Enhance practical confidence and independence

With the continuous accumulation of clinical practicum experience, nursing interns gradually improve their psychological readiness when facing complex clinical scenarios. With appropriate autonomy given by preceptors, they can independently complete full nursing tasks and multiple clinical procedures. In the process of completing entire nursing assignments, they gain a sense of accomplishment, which in turn enhances their practical confidence and independence.

“The first department I rotated to was the ICU. I was really panicked during my first emergency, but after more experience, I no longer felt that way. By the time I was in the emergency department, I was already very proficient in emergency care. During CPR, if my preceptor got tired, I could directly take over compressions, and I could also draw arterial blood gas independently.” (S9)

“My preceptor allowed me to complete the entire set of nursing tasks independently—writing records and performing treatments all by myself, while she just watched by my side. Being able to do everything from start to finish on my own really gave me a sense of accomplishment.” (S11)

##### 3.2.2.3 Improve comprehensive professional literacy

During clinical practicum in a comprehensive teaching hospital, nursing interns not only acquire competencies related to nursing practice but also gain exposure to departmental management and teaching affairs. At the same time, through rich clinical interpersonal interactions, they gradually shed their student mentality and develop their communication and interpersonal skills. This process promotes the comprehensive development of their professional literacy.

“During the period of international hospital accreditation, we had various nursing rounds and drill preparations, which we repeated more than ten times. I learned a great deal from the physical examinations and teaching sessions. In addition, I helped the department organize documents and got to know the teaching and management work of the teaching secretary. Although it was tiring, I really learned a lot.” (S7)

“I used to be very introverted, but now I have become much more outgoing. At the beginning, I didn’t know how to communicate with my preceptor. I always had a student mentality and was afraid to take the initiative to talk to my preceptor, so I just sat there quietly. Now, as long as my preceptor is willing to chat with me, I am also happy to chat with him/her.” (S11)

#### 3.2.3 Perceive professional rewards

During the later stage of clinical practicum, nursing interns gradually perceive multiple rewards from the nursing profession. These include tangible advantages such as employment opportunities, educational requirements, salary, and rest schedules, as well as recognition from patients and preceptors. They also experience the altruistic value of using professional knowledge to help family and friends.

##### 3.2.3.1 Perceive professional advantages

During clinical practicum, nursing interns form subjective perceptions of the professional advantages of nursing, based on their own personal circumstances and the external professional environment. These perceptions are mainly reflected in practical aspects such as employment thresholds, job opportunities, and work-rest schedules. Nursing interns believe that, compared with some other professions, nursing has relatively flexible educational requirements and broader career pathways, along with comparatively stable salary and rest schedules.

“My family believed that healthcare professionals have stable career prospects, and that nursing has lower educational requirements and easier job opportunities compared to clinical medicine. I had a junior college education at that time and was worried about not being able to upgrade to a bachelor’s degree, so I thought nursing was a more secure choice.” (S6)

“Nursing offers good job prospects and decent pay. Several of my friends have similar educational backgrounds to mine, but they studied other majors and couldn’t find jobs, so they ended up working on construction sites with very few days off. Although nursing involves night shifts, we still have regular rest days.” (S8)

##### 3.2.3.2 Gain recognition from others

During their clinical practicum, nursing interns receive positive feedback from patients, preceptors, and others. They also gain positive reinforcement and emotional support through interpersonal interactions. These experiences include immediate encouragement when procedures are successful, as well as the sense of fulfillment and feeling of being needed that comes from building trusting relationships with patients through ongoing care.

“In the past, whenever I successfully inserted an IV catheter, the patient would praise me, and my preceptor would also acknowledge my work. That recognition from others gave me a great sense of accomplishment.” (S2)

“What left a particularly deep impression on me was my time in the gynecology department. I worked with my preceptor to care for that group of patients for four days. Even after I stopped caring for them, they still remembered me. That made me feel that my efforts had been recognized, and I truly experienced the harmony of the doctor-patient relationship.” (S11)

##### 3.2.3.3 Perceive the altruistic value of the nursing profession

Through clinical practicum, nursing interns acquire professional nursing knowledge and health education skills. In addition to serving patients in the hospital, they can also apply what they have learned to their families and friends, providing health consultation and guidance to those around them. They help relatives alleviate the confusion and anxiety caused by illness, and in the process of helping others, they perceive the altruistic value of the nursing profession.

“Whenever I go home on holidays, my family and friends often ask me for advice on professional matters, and I am able to provide them with professional explanations.” (S3)

“During the time when my sister-in-law’s father had liver cancer, he consulted me about many things, including the choice of hospital and doctor, and the location for placing an implantable port. When I came across something I didn’t understand, I would ask the preceptors in my department. Before my mother underwent total knee replacement surgery, she was very anxious. I explained relevant knowledge to her and gave her some examples to reassure her. Later, she had the surgery and is recovering well now. My family also feels that having someone in the medical field at home is very helpful.” (S7)

#### 3.2.4 Deepen professional conviction

During the later stage of clinical practicum, nursing interns' conviction in the nursing profession continuously deepens through their clinical experiences. When faced with setbacks or negative events, they re-evaluate their own abilities and roles through self-reflection and adjustment. In successful experiences such as participating in patient care and life-saving efforts, they genuinely perceive the professional value of nursing work. At the same time, nursing interns gradually shift their perception of nursing from a stable job to a career worth pursuing in the long term, gradually forming career commitment.

##### 3.2.4.1 Reconstruct self-cognition

When nursing interns encounter setbacks and negative events in clinical practice, they undergo dynamic self-cognitive reconstruction and psychological adjustment. Faced with questioning, rejection, or even blame from patients and their families, nursing interns may experience feelings of frustration. However, they are able to gradually move beyond self-doubt and view the events more objectively. Through adjusting their mindset and intensifying hands-on practice, they respond constructively to these challenges, re-evaluate themselves in the face of setbacks, and thereby adapt to the complex clinical work environment.

“When I was drawing an arterial blood gas sample from a patient before and didn’t succeed, as I was preparing to try again, his father suddenly started yelling at me, saying I didn’t know how to do it. I was completely stunned—arterial punctures are inherently difficult anyway … But I didn’t dwell on it too long. I realized it wasn’t my fault; I just need more practice.” (S1)

“There was an aunt in the cardiology department who was very sharp-tongued. She asked my preceptor directly, right in front of me, why she had arranged for a nursing intern to perform the venipuncture on her. After this setback, I made up my mind to improve my technical skills, hoping that patients would not be able to tell whether I was a nursing intern or a registered nurse. During that time, I also specifically practiced venipuncture procedures.” (S7)

##### 3.2.4.2 Recognize professional value

Nursing interns diligently perform standardized nursing procedures in clinical practice and through astute professional observation identify and mitigate clinical risks. In doing so, they gain a tangible sense of professional achievement and come to appreciate the professional essence and value of nursing, dispelling the stereotypical perception that nursing is merely about administering injections and dispensing medications. Meanwhile, their firsthand experience of participating in emergency rescues and witnessing critically ill patients' turn for the better further enhances their perceived professional benefits.

“When helping patients solve practical problems, we feel that this profession is meaningful. Nursing is not just about giving injections and dispensing medications, as outsiders often say. Through observation, we can also reduce the occurrence of accidents, which is great.” (S2)

“During my clinical practicum in the cardiology department, an elderly patient suddenly went into cardiac arrest. My preceptor and I performed resuscitation together, and I participated throughout the entire process. We finally saved the patient, which gave me a strong sense of perceived professional benefits.” (S4)

##### 3.2.4.3 Form career commitment

Nursing interns' perceptions of the nursing profession gradually shift during clinical practice. At the early stage, some interns view nursing merely as a stable job. However, through hands-on clinical experience and exposure to the technical depth and social value of nursing work, their professional perceptions undergo transformation. Positive internship experiences help them break free from stereotypical views of the profession, foster a willingness to engage in nursing as a long-term career, and eventually form career commitment.

“When I first started my clinical practicum, I only thought nursing was a stable job. But after seeing my preceptors use their professional knowledge to relieve patients’ pain and earn recognition from patients and their families, I gradually came to feel that nursing is a career worth pursuing for a lifetime.” (S1)

“Many people think nursing is just giving injections and performing venipuncture. But in reality, nursing requires a high level of technical skill and adequate knowledge, and not everyone is competent for this job. My positive clinical practicum experience has further strengthened my commitment to this profession.” (S6)

## 4 Discussion

The four themes identified in this study reflect, from different dimensions, the perceived professional benefits experienced by nursing interns during the later stage of clinical practicum. From the perspective of positive psychology, this study focuses on nursing interns' active perception of professional benefits. The findings may provide a reference for optimizing clinical teaching and enhancing nursing interns' positive professional experiences.

This study found that support and encouragement from the departmental atmosphere and preceptors help nursing interns alleviate practicum-related stress and improve their clinical practice competence. At the level of the clinical learning environment, a departmental atmosphere characterized by support, respect, and encouragement can stimulate nursing interns' motivation to participate in ward activities (24). This is consistent with the findings of Pedregosa et al. (25), which showed that a clinical environment with a collaborative atmosphere, good interpersonal relationships, and opportunities for professional development helps cultivate nursing interns' critical thinking, communication, and problem-solving skills. In addition, nursing interns reported that the guidance and care provided by preceptors during clinical practicum can both consolidate their clinical operation skills through hands-on teaching and alleviate practicum-related frustration through emotional support, thereby facilitating their personal growth.

Under the current system in China, nursing interns who have not yet obtained their licensure must perform procedures under the supervision of preceptors. This requires preceptors to assume the triple roles of teacher, supervisor, and role model. However, previous studies have pointed out that most European countries have not yet established specific training systems for clinical preceptors, and administrators often give insufficient consideration to preceptorship workload when assigning students (25). Inadequate supervision and insufficient guidance during the preceptorship process can not only hinder nursing interns' skill development and professional growth, but also compromise the quality of patient care (26). Therefore, hospitals should, on the basis of fostering a supportive departmental atmosphere, strengthen the preceptor management system and provide targeted training. These measures aim to ensure standardized supervision, effective guidance, and timely feedback in the preceptorship process.

After repeated clinical training, nursing interns' operational skills improve during the later stage of clinical practicum, which lays a practical foundation for their clinical work after graduation. This is consistent with the findings of Haririan et al. (27) among nursing interns in Iran, which indicated that skill acquisition is highly dependent on hands-on opportunities. A lack of such opportunities can limit the development of operational competence, whereas adequate clinical practice can support nursing interns in transitioning to their professional roles. In addition, existing qualitative studies have largely focused on the early stage of clinical practicum, primarily exploring adaptation challenges such as learning disconnect, negative emotions, and lack of confidence in performing procedures that nursing interns face when first entering clinical settings (28). In contrast, the present study adopts a later-stage perspective, capturing the process through which nursing interns progress from novice to competent in their clinical skills.

Meanwhile, this study was conducted in a tertiary Grade A comprehensive teaching hospital, where the Nursing Department centrally coordinates clinical practicum rotations and assigns registered nurses with preceptor qualifications to provide one-on-one supervision. The hospital offers abundant teaching resources and well-established management protocols. In this environment, nursing interns not only receive systematic and standardized clinical guidance but also have opportunities to participate in ward management and teaching support activities, which facilitate the development of their comprehensive professional literacy.

Traditional nursing education, constrained by limited faculty and resources, has largely emphasized clinical procedures and medication administration, while paying insufficient attention to the cultivation of interns' management and leadership competencies (29). However, leadership cannot be acquired solely through classroom instruction or passive clinical observation; it must be grounded in real clinical settings and developed through hands-on practice (30). Laugaland et al. (31) found in their study of nursing interns in Norwegian nursing homes that when facilities faced staffing shortages and preceptorship was inconsistent, interns were often treated as a workforce rather than learners, with limited access to practice opportunities in nursing management. This suggests that the objective conditions of the practicum setting shape interns' learning content and capacity for professional development. Therefore, it is recommended that hospitals establish robust preceptorship systems when hosting nursing interns. While ensuring sufficient opportunities for hands-on clinical practice, preceptors should appropriately involve interns in ward management and teaching-related tasks. By leveraging authentic clinical scenarios, both clinical skills and leadership capabilities can be simultaneously enhanced, thereby facilitating a smooth transition into the professional nursing role.

Currently, most studies on nursing interns' internship experiences have predominantly focused on examining their subjective feelings within clinical settings (32, 33). Chen et al. (34) explored nursing interns' internship experiences throughout the entire clinical practicum cycle, but their emphasis remained on the internal experiences derived from clinical care and the preceptorship environment. However, they did not specifically target the later stage of clinical practicum, a period when interns approach graduation and career decision-making, to investigate the psychological impact of practical external factors such as employment opportunities and salary expectations. In contrast, the present study specifically examines nursing interns' subjective perceptions of tangible professional rewards and practical gains.

This study found that during the later stage of clinical practicum, nursing interns perceived positive feedback from the nursing profession in terms of employment prospects, compensation, and recognition from others. Some students and their parents believed that, compared with clinical medicine, the nursing profession entailed lower barriers to further education and employment. This may be related to the duration of training: nursing programs typically last 3–4 years, whereas clinical medicine programs in China involve considerably higher economic and time costs, requiring the completion of standardized residency training before one can become a fully licensed physician (35). In addition, nursing interns reported that receiving positive feedback and immediate encouragement from patients and preceptors gave them a sense of achievement and further deepened their perceived professional benefits. Tang et al. (36) pointed out that positive feedback and recognition from patients and preceptors, as external incentives, help enhance nursing interns' sense of achievement and self-worth, enabling them to maintain a positive attitude when facing clinical challenges. Therefore, preceptors should pay attention to nursing interns' emotional experiences during clinical practice and provide timely positive feedback and encouragement (37) to strengthen their perceived professional benefits.

Meanwhile, previous studies on nursing interns' professional identity have largely focused on the facilitating effects of positive experiences on professional identity and the negative impacts of adverse clinical events, yet relatively few have delved into interns' self-coping processes following setbacks (38, 39). Zhang (38) explored nursing interns' professional identity and described the stress and negative feelings they experienced in frustrating situations. However, his study focused on changes in professional identity without further examining the specific coping strategies and psychological adjustment processes that interns employed when facing setbacks. In contrast, the present study revealed nursing interns' behavioral responses when confronted with questioning and refusal from patients and their families. Nursing interns reported that when confronted with questioning or refusal from patients and their families, they experienced frustration but would engage in self-adjustment and intensive practical training to adapt to clinical work. Liu et al. (40) also pointed out that nursing interns commonly experienced a sense of loss after setbacks in clinical procedures, longed for understanding and encouragement, and often adopted strategies such as venting, distraction, and self-reflection to regulate their mindset. Therefore, it is recommended that preceptors provide timely remediation when nursing interns encounter setbacks in clinical procedures, minimize patient and family dissatisfaction, and offer appropriate comfort and encouragement (40). Meanwhile, it is suggested that hospitals, when arranging internship schedules, emphasize standardized training in basic clinical procedures, adaptation to the clinical environment, and communication skills at the early stage, and gradually incorporate complex scenario simulations in the middle and later stages (41), so as to systematically enhance nursing interns' clinical competence.

Through experiences such as identifying problems through professional observation, assisting in risk mitigation, or participating in emergency rescues, nursing interns gain a tangible sense of achievement, which further deepens their perceived professional benefits. Tang et al. (36) pointed out that when nursing interns solve problems using clinical thinking and critical thinking in response to emergencies or challenges, they obtain a significant sense of professional achievement. Meanwhile, clinical internship experiences can deepen nursing interns' understanding of the nursing profession, break down stereotypes, strengthen their willingness to pursue nursing as a career, and gradually form career commitment.

On the one hand, prior to internship, nursing interns' understanding of the nursing profession primarily came from classroom theoretical learning and simulated training in laboratories; even after completing systematic coursework, their professional cognition remained relatively one-sided and superficial (42). On the other hand, clinical internships enable nursing interns to apply theoretical knowledge in authentic clinical settings and, through observing and reflecting on clinical practice exemplars, progressively deepen their understanding of the nursing profession and ultimately internalize their professional conviction (43). On this basis, positive internship experiences further help nursing interns break free from one-sided perceptions of nursing, foster a willingness to pursue nursing as a long-term career, and gradually form career commitment. It is evident that clinical internship serves as a crucial link between theory and practice. It is recommended that hospitals provide nursing interns with more opportunities to manage real and complex cases during the internship training process. Meanwhile, preceptors should guide them to engage in reflective practice through case discussions and reflective diaries, so as to help them construct positive professional perceptions and consolidate their career commitment.

### 4.1 limitations

This study also has several limitations. First, it only included nursing interns from a single tertiary Grade A comprehensive teaching hospital in Hainan Province. The transferability of the findings may be limited by the geographical location and hospital level, and future multicenter studies are recommended. Second, it only focused on nursing interns in the later stage of their clinical practicum. Without a longitudinal comparison across the early, middle, and later stages, the dynamic trajectory of perceived professional benefits throughout the entire clinical practicum process cannot be revealed. Third, the majority of participants in this study expressed an intention to continue working in nursing after graduation, which may have introduced selection bias and response bias. Nursing interns with poorer practicum experiences, those who had experienced transition shock, or those who had already decided to leave the nursing profession might have been unwilling to participate. Although some participants recounted negative experiences during the interviews (e.g., being scolded by patients or physicians, or experiencing clinical procedure failures), most of them viewed such setbacks as opportunities for personal growth and self-cognitive reconstruction. Therefore, the findings of this study mainly reflect the experiences of nursing interns with relatively positive practicum experiences and stable career intentions, and may not adequately capture the perspectives of those who were undergoing severe transition shock or had contemplated leaving the profession. Future studies could optimize the purposive sampling strategy by intentionally including nursing interns with negative career intentions or unfavorable practicum experiences, so as to gain a more comprehensive understanding of perceived professional benefits among nursing interns in the later stage of clinical practicum.

## 5 Conclusions

The later stage of clinical practicum is a critical window for the formation of nursing interns' perceived professional benefits. During this stage, nursing interns gradually develop intrinsic professional identity and professional commitment by obtaining diverse support, strengthening clinical practice competence, and perceiving professional rewards.

Based on these findings, this study offers the following practical recommendations. In terms of practicum planning, hospitals are advised to adopt a phased and progressive model. Specifically, in the early stage, emphasis should be placed on basic procedures and clinical adaptation training. In the middle and later stages, exposure to complex cases and experiential learning through simulation should be gradually increased, with case discussions and reflective journals used to guide nursing interns' practice reflection. In terms of preceptor support, preceptors should provide timely positive feedback and emotional support while ensuring adequate opportunities for hands-on practice. From the perspective of the learning environment, hospitals should foster a collaborative departmental atmosphere, improve the preceptor management system, and enhance teaching quality through targeted training programs. For professional development, nursing interns should be encouraged to participate appropriately in departmental management and teaching affairs, so as to enhance their comprehensive professional literacy in real-world contexts and facilitate their smooth transition to registered nurses. In summary, this study provides a reference for optimizing teaching strategies during the later stage of clinical practicum and improving the support system for clinical internship education in nursing.

## Data Availability

The original contributions presented in the study are included in the article/Supplementary Material, further inquiries can be directed to the corresponding authors.
